# New Onset of Hepatic Steatosis Post-Severe Multisystem Inflammatory Syndrome in Children (MIS-C): A Case Report

**DOI:** 10.3390/ijerph18136961

**Published:** 2021-06-29

**Authors:** Rossella Sica, Serena Pennoni, Laura Penta, Giuseppe Di Cara, Alberto Verrotti

**Affiliations:** Department of Pediatrics, University of Perugia, 06132 Perugia, Italy; serepennoni@hotmail.it (S.P.); laura.penta@ospedale.perugia.it (L.P.); giuseppe.dicara@unipg.it (G.D.C.); alberto.verrottidipianella@unipg.it (A.V.)

**Keywords:** MIS-C, SARS-CoV-2, hepatic steatosis, ultrasonography, transaminases

## Abstract

The emergence of Multisystem Inflammatory Syndrome (MIS-C) following SARS-CoV-2 infection in children and adolescents provided a new diagnostic and management challenge as there is limited knowledge about this condition and its natural history. In existing literature on MIS-C, there are currently no data about long-term outcomes. We report the case of a 14-year-old boy, with no significant past medical history, who presented a condition of multiorgan dysfunction due to MIS-C, after a SARS CoV-2 infection, and subsequent clinical-laboratory signs of hepatic steatosis at short-term follow-up. The case suggests how hepatic steatosis may be a possible sequela following SARS-CoV-2 infection, MIS-C and its medical treatment. Therefore, a close and long-term follow-up is needed to establish the pathophysiology and the evolution of this condition in patients following MIS-C.

## 1. Introduction

Severe acute respiratory syndrome coronavirus 2 (SARS-CoV-2) infection was first identified in Wuhan, China, in December 2019 and then spread rapidly worldwide causing coronavirus disease 2019 (COVID-19) which became the first pandemic of the 21st century by number of deaths. Unlike adults, in which cytokine storm is frequently accompanied by severe respiratory involvement with development of acute respiratory distress syndrome, children with COVID-19 may be asymptomatic or only have mild symptoms. The most prevalent symptom of COVID-19 in pediatric population is fever, followed by cough, upper respiratory tract symptoms, diarrhea and nausea/vomiting. Only a small percentage of children with COVID-19 required Pediatric Intensive Care Unit (PICU) level care or mechanical ventilation [1]. Conversely, in the latter half of April 2020, reports from the UK [2] and Italy [3], followed by the U.S, described a novel syndrome whose features resembled those of known entities such as Kawasaki Disease (KD), toxic shock syndrome (TSS), and macrophage activation syndrome (MAS), which could lead to shock and multiple organ failure, requiring PICU. This novel syndrome is referred to interchangeably as Multisystem Inflammatory Syndrome in Children (MIS-C) associated with COVID 19 or Pediatric Inflammatory Multisystem Syndrome Temporally associated with SARS CoV-2 (PIMS TS). In a case series comparing children and adolescents with MIS-C vs. those with severe COVID-19, MIS-C was distinguished by having more severe cardiovascular or mucocutaneous involvement and extreme inflammation with a higher neutrophil/leucocyte ratio, higher c-reactive protein (CRP) level and more thrombocytopenia than patients with COVID-19 [4]. Initially, it was believed that this syndrome was specifically related to children and adolescents, but reports of cases of multisystem inflammatory syndrome in adults have recently been described, referring to this syndrome as Multisystem Inflammatory Syndrome in Adults Associated with SARS-CoV-2 Infection (MIS-A) [5]. These reports demonstrates that SARS-CoV-2 can cause this clinical manifestation in subjects of any age. The pathophysiology of this syndrome is under intense investigation but so far remains unclear. It is believed that this syndrome results from an abnormal immune response to the virus. Carter et al. performed a serum profiling of MIS-C patients that revealed high levels of interleukin-1β (IL-1β), IL-6, IL-8, IL-10, IL-17, interferon-γ and differential T and B cell subset lymphopenia in the acute phase. Moreover, high CD64 expression on neutrophils and monocytes, and high HLA-DR expression on γδ and CD4+CCR7+ T cells in the acute phase, suggested that these immune cell populations were activated. Antigen-presenting cells had low HLA-DR and CD86 expression, potentially indicative of impaired antigen presentation. All of these features normalized over the resolution phase [6]. In addition, the hyperinflammatory state was found to be different from that in adult severe COVID-19 and KD. Consiglio et al. performed a systems-level analysis of immune cells, cytokines and antibodies in the blood of children presenting with MIS-C as compared to children with mild SARS-CoV-2 infection and children with KD. They noticed that several details of the hyperinflammatory state in children with MIS-C were different from the hyperinflammation seen in adults and children with acute SARS-CoV-2 infection and that of children with KD as well. In particular, they found that IL-17 is most important in KD but it is significantly lower in MIS-C patients, indicating a difference in the underlying immunopathology [7]. Clinical features of MIS-C are variable and characterized mostly by persistent fever; mucocutaneous symptoms including conjunctivitis and rash; gastrointestinal symptoms, including vomiting, abdominal pain, and/or diarrhea; cardiac abnormalities such as myocardial dysfunction, myocardial infarction with increased serum concentrations of troponin and pro-B-type natriuretic peptide (proBNP), coronary artery dilation or aneurysm, arrhythmia and a compromised hemodynamic status which can lead to acute renal and hepatic failure; neurologic findings including headache, irritability and encephalopathy; and coagulopathy. On 13 May 2020, the Center for Disease Control (CDC) issued a case definition specifying that the patient should be <21 years old, have fever, with laboratory evidence of inflammation (including, but not limited to, 1 or more of the following: an elevated CRP, erythrocyte sedimentation rate, fibrinogen, procalcitonin, D-dimer, ferritin, lactate dehydrogenase, IL-6, elevated neutrophils, reduced lymphocytes and low albumin level), and evidence of clinically severe illness requiring hospitalization, with multisystem (>2) organ involvement (cardiac, renal, respiratory, haematological, gastrointestinal, dermatological or neurological), in the absence of an alternative plausible diagnosis, as well as evidence of SARS-CoV-2 infection or exposure [8]. In existing pediatric literature on MIS-C, only short-term outcomes were reported [9], as there are currently no published reports detailing long term outcomes in these patients.

## 2. Case Report

**Figure 1:**
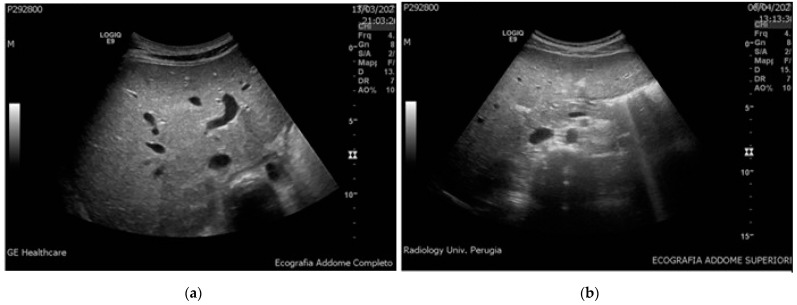
(**a**) Ultrasonography of the abdomen performed at discharge demonstrating normal dimension of the liver and a homogenous pattern. (**b**) Ultrasonography of the liver performed during follow-up demonstrating hepatomegaly and a hyperechoic liver, suggestive of hepatic steatosis.

On 1 March 2021, a 14-year-old boy was admitted to the Pediatric Emergency Department with compromised general status. He was found to be alert and oriented, febrile with 38.2 °C, tachycardic (118/beats/min), hypotensive (60/40 mmHg), saturating 100% on room air breathing 42 breaths per minute. Upon clinical examination, he was found to be dehydrated, with jaundiced skin, capillary refill 3–4 s, cold extremities with feeble peripheral pulses, palmoplantar erythema and edema, diffusely painful abdomen and palpable hepatosplenomegaly. He was in a condition of multiorgan failure: acute kidney injury, hepatosplenomegaly with increased inflammatory markers, in particular of IL-6 (144.6 pg/mL) and compromised hemodynamic status with reduction of left ventricular ejection fraction. We also noticed elevated values of alanine aminotransferase (ALT) (139 UI/L), aspartate aminotransferase (AST) (102 UI/L) and indices of cholestasis. Given the family history of SARS-CoV-2 infection and the positivity of SARS-CoV-2 IgG from serum, he was diagnosed with MIS-C. The patient’s compromised hemodynamic status required vasoactive support at the Cardiac Intensive Care Unit for the first 3–4 days of hospitalization. He was treated with intravenous immunoglobulin (2g/kg over 48 h) and pulse Methylprednisolone (10 mg/kg a bolus twice a day for 3 days and then progressively reduced). In consideration of the elevation of D-dimer (5734 ng/mL), a prophylactic antithrombotic therapy was started with low-molecular-weight heparin and aspirin (3 mg/kg/day). During hospital course, we noticed an improvement of his general conditions, with return to clinical baseline and progressive normalization of laboratory alterations, particularly of ALT and cholestatic indices. Ultrasonography of the abdomen was performed prior to discharge, and we found no abnormalities (Figure 1). He was discharged at day 14 with indication to continue therapy with Aspirin and Prednisone per os, in progressive decalage according to the tapering strategy.

**Figure 2:**
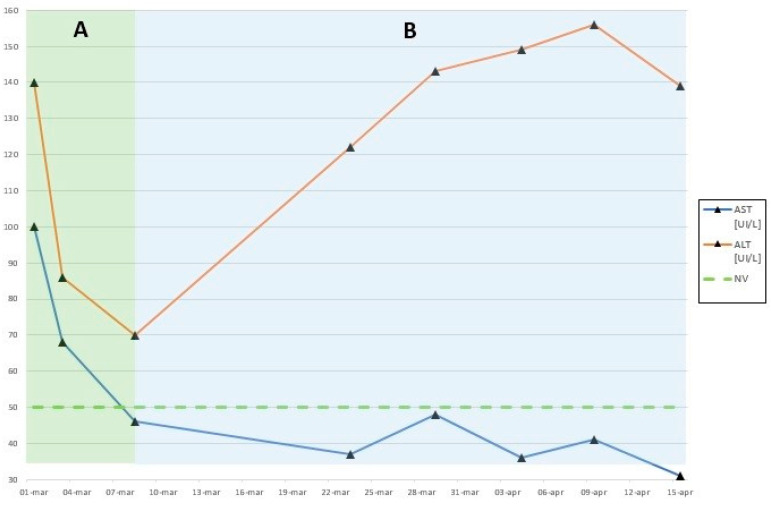
Graphic representation of the ALT and AST levels over time which demonstrates the decrease in ALT and AST during hospitalization (**A**) and then a new rise of the ALT with stable low AST levels during follow-up visits (**B**).

Following discharge, the patient was re-evaluated in a follow-up visit program (physical examination, laboratory tests and echocardiography) during which we started to taper off prednisone (until 1 mg/Kg/day). On 23 March, at the first follow-up visit, the patient was found to be in good conditions, without particular findings on the physical examination. The laboratory tests were normal except for the elevation of ALT (122 UI/L). On the second follow-up visit, 30 days following the onset of MIS-C, the patient was completely asymptomatic but clinical assessment revealed palpable hepatomegaly (2 cm below the costal margin) and further elevation of ALT (143 UI/L) on laboratory testing. AST, serum bilirubin and all the other lab parameters were normal. Ultrasonography of the abdomen was performed and revealed that hepatomegaly was secondary to hepatic steatosis (Figure 1), with absence of pre-existing obesity (BMI 19.2) and signs of Nonalcoholic Fatty Liver Disease (NAFLD). Moreover, any other existing or previous inflammatory pathology was excluded. The following follow-up visits, laboratory tests (Figure 2) and ultrasonographies of the abdomen showed data suggesting hepatic steatosis as well. We did not introduce a drug therapy but we started a lifestyle approach including dietary improvements and increasing physical activity. Our patient was referred to a Pediatric Gastroenterologist. Currently, the patient is in good general clinical condition, with an optimal recovery of cardiac and renal function and not on drug therapy, as steroid therapy was withdrawn during the follow-up visit program. Nevertheless, our patient still has hepatic steatosis which was also confirmed by ultrasonography of the abdomen performed during the most recent follow-up visit on 27 May.

## 3. Discussion

SARS-CoV-2 may cause a systemic disease with possible involvement of other organs besides the respiratory system, including the liver, because of ubiquitous distribution of the main viral entry receptor, namely angiotensin converting enzyme 2 (ACE2). In particular, the virus spike protein binds ACE2 to gain cell entry and transmembrane serine protease 2 (TMPRSS2) and paired basic amino acid cleaving enzyme (FURIN) are also important for infection. Therefore, the expression of these receptors provided early clues for putative hepatic permissive cells [10]. A study of Pirola et al. demonstrates that gene expression levels for ACE2 are highest in cholangiocytes compared with alveolar type 2 cells, followed in turn by sinusoidal endothelial cells and hepatocytes, thus supporting the possibility that SARS-CoV-2 may cause direct liver injury by viral cytopathic effect. The cytopathic effect may be due to a direct mechanism such as lysis or by necrotic and/or apoptotic effects. Furthermore, the expression pattern in cell clusters associated with numerous active immune pathways, for example, inflammatory macrophages, natural killer cells, plasma cells, mature B cells and cells of the liver endothelial microenvironment, opens the possibility of SARS-CoV-2 immune-mediated liver damage [11]. Therefore, the pathophysiology of the liver involvement in COVID-19 and MIS-C is still unclear, but it is believed that the injury might be caused by hepatocellular infection with direct cytopathic effects of SARS-CoV-2 or an immune-mediated response with inflammatory damage. In addition, hypoxic/shock-related circulatory compromise, endothelial dysfunction, microthrombi formation and drug-induced liver injury (e.g., corticosteroids, lopinavir, ritonavir, tocilizumab and remdesivir) may be involved [12]. COVID-19-associated liver injury is defined as any liver damage occurring during disease course and treatment of COVID-19 patients, with or without pre-existing liver disease [13] and typically leads to a temporary moderate elevation of liver tests without significant hepatic synthetic function impairment. Patients with SARS-CoV-2 infection and elevated ALT are at risk of a more severe disease course including longer hospitalization and ICU stays [14]. Patients with greater elevations in serum ALT levels often have high levels of CRP, which is synthesized by the liver, D- dimer, ferritin and IL-6. IL-6 is produced by monocytes, macrophages and T cells in response to activation of the innate and adaptive immune system, and is the most important driver of CRP production, and high IL-6 levels are associated with liver injury in COVID-19 [10]. Hepatitis is also common in children with MIS-C, and it is associated with a more severe presentation of the syndrome [15]. COVID-19-associated hepatocellular injury is characterized by moderate steatosis, lobular and portal inflammation, apoptotic and/or necrotic foci and elevation of plasma ALT and AST [16]. Microvesicular and macrovesicular steatosis have been observed in liver autopsies of COVID-19 patients who presented with SARS-CoV-2 infection and, in some cases, SARS-CoV-2 hepatocellular infection has been proven [16,17]. Induction of host de novo lipogenesis might be crucial for SARS-CoV-2 life cycle as enhanced de novo lipogenesis could supply the virus with sufficient amounts of lipids to generate the vesicular systems required for viral replication and exocytosis [16]. Moreover, steatosis might be caused by SARS-CoV-2 induced mitochondrial dysfunction and Endoplasmic reticulum (ER) stress, which can induce de novo lipogenesis [16,18]. There are observations that suggest that SARS-CoV-2 affects mitochondrial activity, for instance, mitochondrial crista abnormalities were found in liver specimen of COVID-19 patients [17]. In addition, electron microscopy examinations proved SARS-CoV-2 hepatocellular infection and reported a pathological ER dilatation in infected hepatocytes, which is the most probable cause of ER stress [17]. In addition, the coronavirus S protein might play a key role in ER stress induction [19]. SARS-CoV-2 infection might also activate mTOR, inducing de novo lipogenesis and eventually inhibiting autophagy (as a mechanism of viral degradation) and facilitating viral escape from the immune system [16]. Moreover, significantly increased mTOR activity has been revealed upon IL-6 stimulation [20], and IL-6 is one of the most important cytokines involved in MIS-C. Additionally, it is important to differentiate hepatic lipid accumulation as a result of SARS-CoV-2 infection and MIS-C from pre-existing NAFLD, which has been shown to increase the risk for poor outcome [21]. Therefore, we hypothesize that hepatic steatosis in our patient might be caused by SARS-COV-2 infection, COVID-19 and MIS-C. We do not exclude that liver damage and consequent steatosis might be caused by an immune-mediated inflammatory response to the virus or by hypoxic/shock-related circulatory compromised status, endothelial dysfunction and/or microthrombi formation. Furthermore, drug-induced liver damage should not be excluded. In a retrospective chart review of a cohort of children treated with a multidisciplinary approach and consensus-driven algorithm, the short-term outcomes of these patients were found to be generally favorable [9]. However, in existing literature on MIS-C, there are no data concerning the mid-long-term outcomes of these patients. Although much of the discussion around MIS-C and its outcomes has centered on cardiac manifestations and sequelae, such as left ventricular (LV) dysfunction and coronary aneurysms, providers must be aware of the hepatic damage which might be caused either by SARS-CoV-2 infection and immune-mediated response. We suggest that close follow-up for these patients would be needed to assess the evolution of hepatic damage following MIS-C.

## 4. Conclusions

The emergence of MIS-C following SARS-CoV-2 infection in children and adolescents provided new diagnostic, therapeutic and follow-up challenges as there is limited knowledge about this condition and its natural history. The hepatic consequences of SARS- CoV-2 infection are due to possible direct cytopathic effect, systemic inflammation and immune dysfunction and are an important component of both COVID-19 and MIS-C. Moreover, pre-existing NAFLD should be distinguished from hepatic lipid accumulation as a result of SARS-CoV-2 infection and MIS-C. Currently, there are no data reporting mid-long-term outcomes in patients following MIS-C. We report a case with an excellent cardiac and renal outcome but with a new onset of hepatic steatosis. New onset of hepatic steatosis might be one of the sequelae following SARS-CoV-2 infection, MIS-C and its treatment, mainly the prolonged use of corticosteroids. Furthermore, we suggest that hepatic steatosis after COVID-19 and MIS-C might be underdiagnosed as few patients do undergo abdominal ultrasonography monitoring. Diagnosis of hepatic steatosis is easily accessible through an abdominal ultrasound, which is a low-cost, non-invasive examination. We suggest that it can be very useful to identify this potential damage early. Therefore, more studies are needed to establish the pathophysiology and the evolution of this condition in patients following MIS-C. We suggest that long-term follow-up is needed and would be essential to define the late outcomes of these patients.
